# Cellular metabolism as a basis for immune privilege

**DOI:** 10.1186/1476-8518-4-1

**Published:** 2006-03-17

**Authors:** M Karen Newell, Elizabeth Villalobos-Menuey, Susan C Schweitzer, Mary-Ellen Harper, Robert E Camley

**Affiliations:** 1 The Institute for Bioenergetics, University of Colorado at Colorado Springs, Colorado Springs, CO 80933-7150, USA; 2 Department of Biochemistry, Microbiology and Immunology, Faculty of Medicine, University of Ottawa, Ottawa, Ontario, Canada

## Abstract

We hypothesize that the energy strategy of a cell is a key factor for determining how, or if, the immune system interacts with that cell. Cells have a limited number of metabolic states, in part, depending on the type of fuels the cell consumes. Cellular fuels include glucose (carbohydrates), lipids (fats), and proteins. We propose that the cell's ability to switch to, and efficiently use, fat for fuel confers immune privilege. Additionally, because uncoupling proteins are involved in the fat burning process and reportedly in protection from free radicals, we hypothesize that uncoupling proteins play an important role in immune privilege. Thus, changes in metabolism (caused by oxidative stresses, fuel availability, age, hormones, radiation, or drugs) will dictate and initiate changes in immune recognition and in the nature of the immune response. This has profound implications for controlling the symptoms of autoimmune diseases, for preventing graft rejection, and for targeting tumor cells for destruction.

## Review

The immune system, a complex organization of cells, tissues and organs, serves to protect us from potential harm. Extraordinary advances in our understanding of the immune system have been made in the last hundred years, especially since the discovery of T and B lymphocytes [1]. Nonetheless, fundamental questions remain unanswered. One of these unanswered questions concerns the nature of "immune privilege". It is widely accepted that certain tissues (brain, eye, ovary, testes) interact differently with the immune system compared to most other tissues. These tissues are commonly termed "immune privileged" [2], however the basis for the privilege is unknown. The purpose of this report is to suggest a mechanism that accounts for immune privilege.

We recognize that immune privilege is a topic of ongoing discussion. For example, the role of FasL, Transforming Growth Factor beta (TGF-beta), IL-4, and IL-10, among others, have been widely discussed [3,4]. Some recent work relating the cell surface expression of FasL with metabolic intermediates, including cyclooxygenase-2, is consistent with both our hypothesis as discussed below and the involvement of FasL in immune privilege[5].

Recognition of antigen by T lymphocytes (T cells) and the subsequent activation of the T cell, are crucial steps within the immune response and immune recognition. Naïve T-cells require at least two signals for activation. These are recognition of antigens in Major Histocompatibility Complex-encoded (MHC) molecules [6], and a co- stimulation signal [7-9] provided by the B7/CD28 family members or other co-stimulatory molecules such as Fas (CD95) or CD40. Previously activated T cells can be reactivated by co-stimulation alone [10,11]. In the absence of activation, T-cells do not respond to self tissue, i.e. the T cells tolerate the tissue. The consequences of T cell activation include: 1. destruction of damaged cells [12,13], or 2. repair of damaged cells by promoting regeneration either directly or indirectly [14-16].

## The connection between cellular metabolism and immune privilege

We hypothesize that energy metabolism has a primary influence on the presence or absence of both T cell activation signals and thus regulates "immune privilege". What do we mean by energy metabolism? In non-dividing cells, mitochondria normally provide over 90% of cellular ATP. The details of this energy storage process are complex, but there are key parameters that control ATP production. These include: a proton gradient across the inner mitochondrial membrane that contributes to an electrochemical "proton motive force" across the membrane, an electron transport chain along the inner membrane, and respiratory complexes within the inner membrane. Oxygen complexes are used to facilitate the electron flow, with the terminal reaction involving the reduction of molecular oxygen to water. Thus normal by-products of energy production are reactive oxygen intermediates (ROI).

We have observed that the choice of fuel (glucose and/or lipid) used for mitochondrial metabolism, is part of a metabolic behavior that regulates the interaction of the cell with other cells, including cells of the immune system [17,18]. We propose that there are at least two metabolic base states. Immune-sensitive cells use carbon atoms derived primarily from glucose for fuel in the mitochondria, exhibit relatively high mitochondrial membrane potential, may have increased levels of cell surface MHC, are easily damaged by free radicals (including excessive reactive oxygen intermediates), and may show increased levels of cell surface co-stimulatory molecules. Immune-sensitive cells are thus defined as cells that interact readily with the immune system. This can include homeostasis [19], regenerative growth nurtured by the immune system [15,16], or immune-induced death of infected or damaged cells [12]. In contrast, immune-privileged cells preferentially use lipids for fuel, have a lower mitochondrial membrane potential, are less likely to express cell surface MHC molecules, are less easily, or are more resistant to, damage caused by free radicals, and have relatively lower levels of co-stimulatory molecules.

As evidence for this idea, we observe that some cells predominantly use carbon atoms derived from glucose as fuel in the mitochondria (leukocytes, hepatocytes, epithelial cells, regenerating tissues, and many drug sensitive tumors) [20-23] while other types of cells (brain, pancreatic beta cells, muscle, eye, drug-resistant tumors) can use glucose *or *lipids. The first group of cells has been shown to be readily recognized by the immune system, while the second group is considered immune-privileged [2].

## Factors affecting metabolism and immunological signals

Not surprisingly, control of the first and second activation signals for the immune-sensitive cells may also be metabolically based. For example, it is known that MHC class II cell surface expression, a requirement for signal one, increases during inflammation and inflammation correlates with local changes in metabolism [24]. We propose that fuel consumption and energy production in the cell control the production of free radicals. The existence of intracellular free radicals, in turn, is associated with changes in the level of MHC class II expressed on the cell surface [24] and with modifying or inducing the second signal [25]. Thus cellular metabolism may affect how, the immune system "sees", recognizes and responds to, a given cell or tissue.

There are a wide variety of extrinsic factors – chemotherapeutic agents, anti-metabolites, insulin, glucose, fatty acids, nerve (and other) growth factors, oxidative stressors (hypoxia, hyperoxia), and low intensity microwaves – that are known to alter the metabolic strategy of the cell. In each case there is a corresponding change in the immunological signals the cells presents to T lymphocytes. The pharmacologic mechanism of many drugs is based on interfering with cellular metabolism [26]. We, and others, have found that chemotherapeutic agents, including methotrexate and adriamycin, modify the levels of cell surface expression of the costimulatory molecules B7.1 (CD80), B7.2 (CD86), and Fas (CD95) on drug-treated cells [17,26]. In most cases, the level of B7.1 is at least doubled.

**Figure 1:**
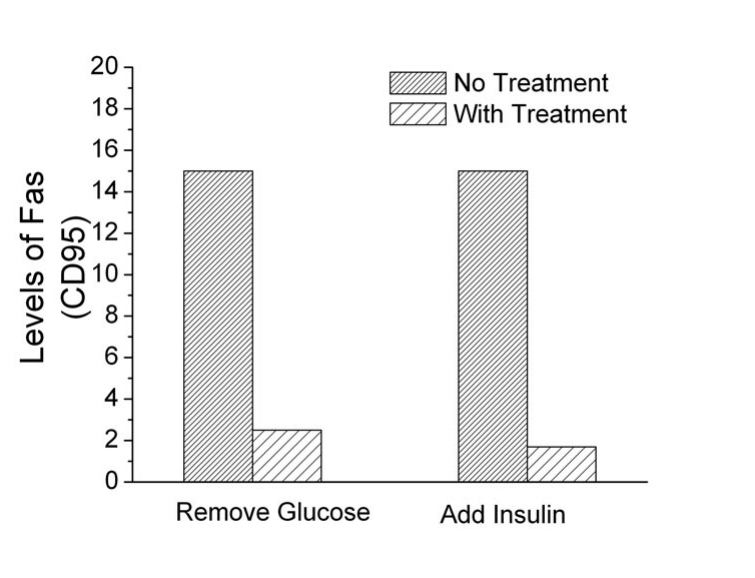
**Metabolic modification of cell surface Fas expression**. Changes in expression of Fas caused by removing glucose or adding insulin to the culture medium. The glucose is removed by incubating HL60 (human promyelocytic leukemia) cells in glucose free RPMI with the addition of 5 mM 2-Deoxyglucose. When cells were incubated with insulin, normal RPMI medium conditions were used with the addition of 100 μg/mL of insulin. The above conditions represent a 24 hour treatment period. This data is representative of five separate experiments. Each of the experiments showed the same general trends, however the experiments were done at different times and because the intensity of the fluorochromes varies with time, this makes direct statistical comparisons suspect. The level of Fas was detected using PE-conjugated anti-humanFas (CD95) antibodies (Pharmingen, California) and measured using a Coulter Elite Epics Flow Cytometer (Coulter, Hialeah, Florida) and FlowJo analysis software (Tree Star, Inc, Oregon).

A surplus or a deficit of specific nutrients also affects metabolism. For example, addition of insulin can reduce levels of Fas ten-fold [27]! Similarly, cells incubated in medium where glucose has been removed, also show substantial reductions in cell surface Fas, (Figure 1). In contrast, when glucose levels are increased above normal levels, cell surface Fas expression increases [28]. These data provide direct evidence that changes in metabolism can make a cell less visible to the immune system and thus confer immune privilege.

Additional support for our model is seen with immune privileged cells. Many types of cancer cells are effectively immune privileged. Among cancer cells, melanoma is well-characterized as an immunologically silent tumor [28]. Because melanoma cells have been shown to preferentially utilize fat for fuel [18], their low immunogenicity is consistent with a model in which immune privilege correlates with the ability to use, or even choose to use, fat as a source of fuel.

**Figure 2:**
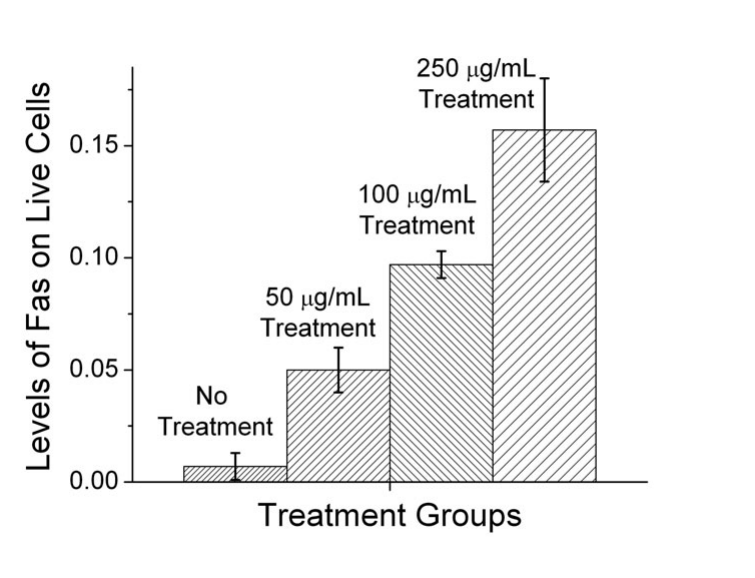
**Inhibition of CPT induces increased cell surface Fas expression**. Levels of cell surface Fas on B16F1 melanoma cells in cultures with different concentrations of Etomoxir for 24 hours. Etomoxir blocks the mitochondria from using carbons from fat as fuel. Fas levels, normally low in melanoma cells, rise in the cells treated with Etomoxir. At a concentration of 500 μg/mL all the cells died. The level of Fas was detected using PE-conjugated anti-mouse Fas (CD95) antibodies (Pharmingen, California) and measured using a Coulter Elite Epics Flow Cytometer (Coulter, Hialeah, Florida) and FlowJo analysis software (Tree Star, Inc, Oregon).

An important question is whether interfering with the process of burning fat removes the immune privilege, thus making melanoma cells more visible to the immune system. We demonstrate such a change in immunogencity by treating melanoma cells with etomoxir, an inhibitor of carnitine palmitoyl transferase (CPT) [29]. CPT is required for the transport of fatty acyl residues into the mitochondria, thus, treating cells with etomoxir essentially blocks the ability of the mitochondria to use carbon atoms derived from fatty acids [29]. When B16F1 melanoma cells are incubated in medium containing 50 μg/mL, 100 μg/ml, and 250 μg/ml of etomoxir, we observe a dose dependent increase in cell surface Fas (Figure 2). The expression of cell surface Fas increases the visibility of the melanoma cells to cells of the immune system, particularly to cells expressing cell surface Fas ligand (FasL), because these cells could potentially induce apoptosis. A similar increase in Fas expression has been observed in other cell lines, including L1210 and its drug resistant counterpart L1210DDP, when exposed to etomoxir.

Increasing a cell's visibility to the immune system can result in a variety of immune responses. These include the release of different types of cytokines that could differentially promote either growth or death of the recognized tissue. In addition, signals resulting from cell to cell contact may also be involved in a cells decision to either grow or to undergo apoptosis. Members of the nerve growth factor/nerve growth factor receptor families, including Fas and Fas Ligand, are well established mediators of both growth and death signals [15,16,30,31].

## Metabolic states and uncoupling proteins

We suggest that uncoupling proteins (UCPs) are a part of the mechanism controlling the change from one metabolic strategy to another. Uncoupling proteins are a family of molecules, first described in brown adipose tissue, that function as a metabolic switch [32,33]. These proteins have been shown to produce the following metabolic changes: dissipation of the mitochondrial proton gradient, thermogenesis, in the case of UCP 1 [32], lowering of mitochondrial membrane potential; induction of a metabolic shift to fatty acids as a carbon source of fuel in mitochondria [18]; promotion of high rates of glucose utilization in the cytosol and increased oxygen consumption in the mitochondria and protection from reactive oxygen intermediates. Clearly, there is a striking similarity between the known changes in metabolic activity produced by uncoupling proteins and the metabolic features associated with immune privilege.

In addition to the evidence described above, other recent studies also support this model. The characterization of two distinct cellular metabolic strategies has recently been used to distinguish drug-sensitive from drug-resistant tumor cells [18]. Furthermore, several studies have documented differences between the cell surface expression of important immune molecules (such as MHC class I and II, Fas, and B7 family members) on drug-sensitive compared to drug-resistant tumor cells [17,18,34]. The concept of two basal metabolic states that affect immune recognition is further supported by observations that drug-sensitive cells expressing immune molecules die by apoptosis more readily than drug resistant cells. The activity of uncoupling proteins, along with the existence of distinct metabolic states, may provide the causal link between these observations.

**Figure 3:**
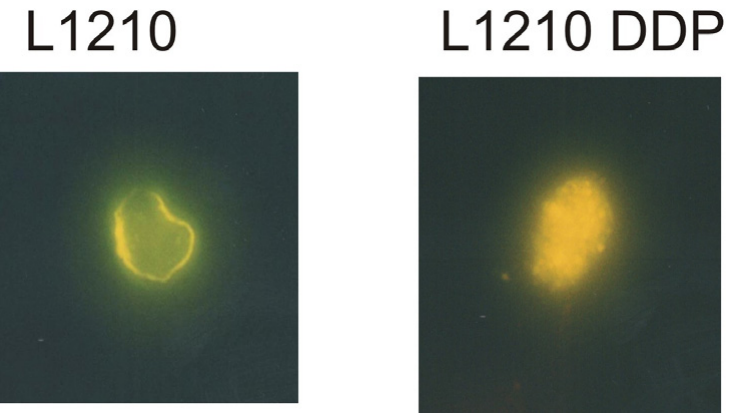
**Confocal Microsopy of L1210 (rapidly dividing cells) and L1210 DDP (slowly dividing cells) showing distribution of UCP2**. The slowly dividing cells have substantial UCP2 within the cell and also have little to no cell surface Fas. In contrast, the rapidly dividing cells have UCP2 on or near the cell surface and have significant levels of cell surface Fas.

If uncoupling proteins play a critical role in creating the two distinct metabolic states, one would expect significant differences in the behavior of UCP in cells with different metabolic states. To observe any differences in the distribution of UCP2, we transfected metabolically distinct cells with green fluorescent protein labeled UCP2 (Figure 3). Confocal micrographs of L1210 cells, (predominantly use glucose for fuel), and L1210 DDP cells, (readily burn fat for fuel), show a clear difference in the distribution of UCP2. L1210 DDP cells have substantial UCP2 within the cell, in contrast to L1210 cells that have detectable UCP2 only on or near the cell surface. L1210 are rapidly dividing cells and L1210 DDP are slowly dividing cells. The slowly dividing cells have no cell surface Fas [17]. In contrast, the rapidly dividing cells have significant levels of cell surface Fas. Taken together these data suggest a correlation between subcellular distribution of UCP2 and cell surface Fas expression.

## Immune privilege and the danger model

We comment on the connection between our hypothesis and the Danger model [35], a paradigm that argues that intrinsic or extrinsic stresses on a cell produce a danger signal, which results in the expression of co-stimulation molecules. Our model of immune privilege suggests that the Danger hypothesis could apply for both immune-privileged and immune-sensitive tissues. However, in immune-privileged cells, there is a mechanism to reduce the likelihood of the Danger signal, resulting in a reduced capacity for co-stimulation.

Extending the Danger model, we suggest a change in metabolism can lead to a change (either an increase or a decrease) in the number of free radicals in the cell and this, in turn, leads to a change in the level of the co-stimulatory signal and MHC class II expression. Recent work demonstrating that high levels of ambient glucose result in an increase in intracellular free radicals, e.g. reactive oxygen [27], supports a portion of this hypothesis. We, and others, have directly shown that reactive oxygen impacts the expression of both B7 family members and Fas (CD95) [36]. Clearly, these data provide substantial evidence for the link between metabolism and immune recognition.

As a point of clarification, because all cells use both glucose and lipids, it is not the choice of fuel, alone, which determines whether a cell is immune-privileged or not. Our model proposes it is the switch from using carbon atoms derived from glucose as the primary fuel in the mitochondria, to using lipids as the primary fuel, accompanied by the shift in metabolic parameters described above, that results in a cell being less visible to the immune system.

## Conclusion: Implications of the model

This connection between cell metabolism and the immune system is profound. If we can change how the immune system recognizes a cell, we may be able to direct the immune system to ignore, destroy, repair, or regenerate the recognized cell. This is, for example, especially important for controlling autoimmune diseases such as multiple sclerosis (MS) and rheumatoid arthritis where the goal is to prevent the immune system from attacking our own tissue. In fact, our model could explain the observation that reducing caloric intake lessens autoimmune symptoms [37,38]. Similarly, inducing different changes in cellular metabolic activity might provide a strategy for destruction of tumor cells. Finally, changes in metabolism could produce changes in signal one and signal two, which could lead to repair and regeneration of neurons. This could be very important in helping stroke victims or people with spinal cord injuries.

If our hypothesis is correct it allows some speculation regarding our inability to regenerate most organs and limbs. It is known that immune-privileged tissues (which do not normally express cell surface MHC) do not regenerate easily. It is interesting to note that the appearance of MHC on the phylogenetic tree occurred in the evolutionary period between newts (which can regenerate) and nurse sharks (which can't regenerate). We note that the nurse shark was also the first to exhibit thermogenesis in the brain and coincidentally to express MHC. Did we acquire specificity in the immune system and warmth, and in trade, lose the ability to regenerate tissues without MHC or antigen?

## Testing the hypothesis

We have proposed that different metabolic base states, which have distinct metabolic strategies, determine whether a cell has immune privilege or not. One way to test this idea is to create immune-privileged cells by transfecting the gene encoding an uncoupling protein accompanied by an inducible promoter. This process would allow the expression of UCP to be turned on and off on cue – effectively creating immune-privileged cells when UCP is on. We could then stress cells (with and without UCP) and see if the levels of co-stimulatory molecules are lower on the immune-privileged cells as compared to the normal cells. Extending this approach, we could test the ability of transfected or non-transfected cells to present antigens to antigen-specific T cells. If the hypothesis is correct, the immune-privileged cells will be less capable of activating T cells.

There are also *in vivo *tests for the hypothesis. For example, we know that immune mediated rejection is the key problem for successful transplants. As described above, we could transfect stem cells with the gene for UCP, theoretically creating an immune-privileged stem cell. Our model would predict that if these cells were transplanted into an allogeneic recipient, rejection would not occur.

In conclusion, we propose an intimate connection between cellular energetics and how the immune system responds to an individual cell. If true, this could have a major impact on the treatment of many diseases ranging from cancer to multiple sclerosis.

## Competing interests

The University of Colorado and the University of Vermont hold patents (licensed to Newellink USA Inc.) pertaining to metabolism and the immune response.

## Authors' contributions

This paper is distinct because it is a theoretical opinion paper. However, each author contributed uniquely to the manuscript. Author 1, MKN, provided the conceptual framework for the hypothesis presented in the paper; Author 2, EVM, performed the experiments described in Figures 1 and 2; Author 3, SCS, contributed her findings on the impact of exogenous and endogenous fatty acids on MHC expression as well as providing her expertise in lipid metabolism; Author 4, M-E. H., transfected tumor cells with GFP-flagged UCP2 and provided the confocal micrographs, Figure 3; Author 5, REC, participated in the development of the hypothesis, discussions of the hypothesis, and drafts of the manuscript.
